# Supplementary material for: Snail Transcriptionally Represses Brachyury to Promote the Mesenchymal-Epithelial Transition in Ascidian Notochord Cells
Source: Int J Mol Sci. 2024 Mar 18;25(6):3413. doi: 10.3390/ijms25063413 (PMC10970311; doi:10.3390/ijms25063413)
Supplement: Supplementary file 1 [file ijms-25-03413-s001.zip › ijms-2890415-supplementary.pdf]

International Journal of *Molecular Sciences*

MDPI

Article

Snail Transcriptionally Represses *Brachyury* to Promote the Mesenchymal-Epithelial Transition in Ascidian Notochord Cells

Bingtong Wu [1], Xiuke Ouyang [1], Xiuxia Yang [1,2,3,*] and Bo Dong [1,2,3]

[1] Fang Zongxi Center for Marine EvoDevo, MoE Key Laboratory of Marine Genetics and Breeding, College of Marine Life Sciences, Ocean University of China, Qingdao 266003, China; wubingtong@stu.ouc.edu.cn (B.W.); ouyangxiuke88@163.com (X.O.); bodong@ouc.edu.cn (B.D.)

[2] Laboratory for Marine Biology and Biotechnology, Qingdao Marine Science and Technology Center, Qingdao 266237, China

[3] MoE Key Laboratory of Evolution and Marine Biodiversity, Institute of Evolution and Marine Biodiversity, Ocean University of China, Qingdao 266003, China

* Correspondence: xxyang@ouc.edu.cn

Citation: Wu, B.; Ouyang, X.; Yang, X.; Dong, B. Snail Transcriptionally Represses *Brachyury* to Promote the Mesenchymal-Epithelial Transition in Ascidian Notochord Cells. *Int. J. Mol. Sci.* **2024**, *25*, x. https://doi.org/10.3390/xxxxx

Academic Editor: Matthias Nees

Received: 9 February 2024
Revised: 11 March 2024
Accepted: 13 March 2024
Published: 18 March 2024

Copyright: © 2024 by the authors. Submitted for possible open access publication under the terms and conditions of the Creative Commons Attribution (CC BY) license (https://creativecommons.org/licenses/by/4.0/).

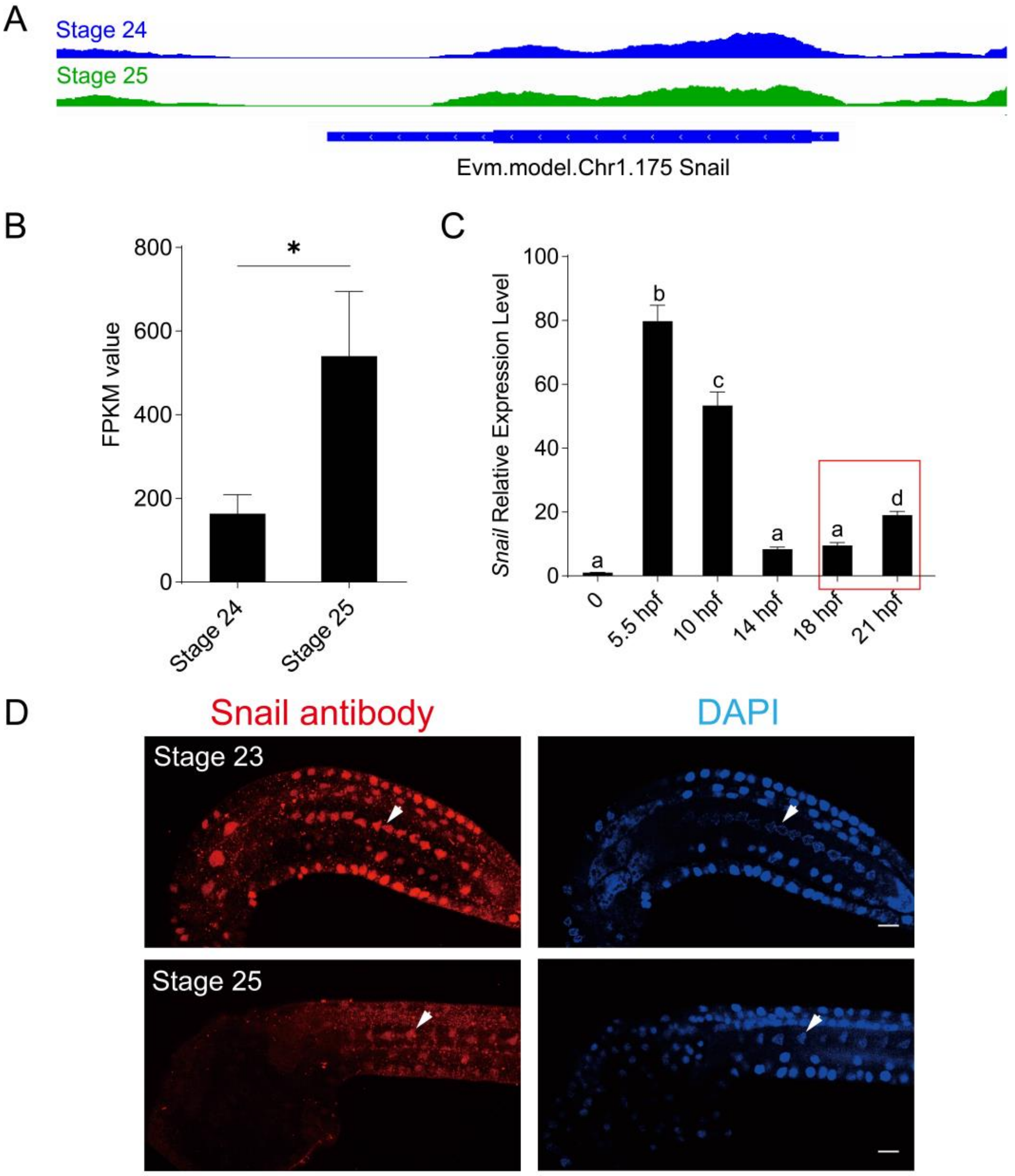

Figure S1. Expression pattern and subcellular localization of *Ci-Snail*. (**A**) ATAC-seq analysis showed that *Ci-Snail* expression is activated with open chromatin region during MET process. (**B**) Smart-Seq of notochord cells showed that *Ci-Snail* expression increased significantly during MET process. "*" indicates significant difference ($p < 0.05$). (**C**) *Ci-Snail* expression levels at different developmental periods. Red box indicates that Snail expression was reactivated during MET process. Significance difference is marked by letters (a-d). The identical letter indicates that the difference is not significant, whereas the different letter indicates the difference is significant. (**D**) Immunofluorescence assay showed *Ci-Snail* expressed in the notochord nucleus during MET process. White arrowheads represent notochord nucleus. The scale bars represent 10 μm.

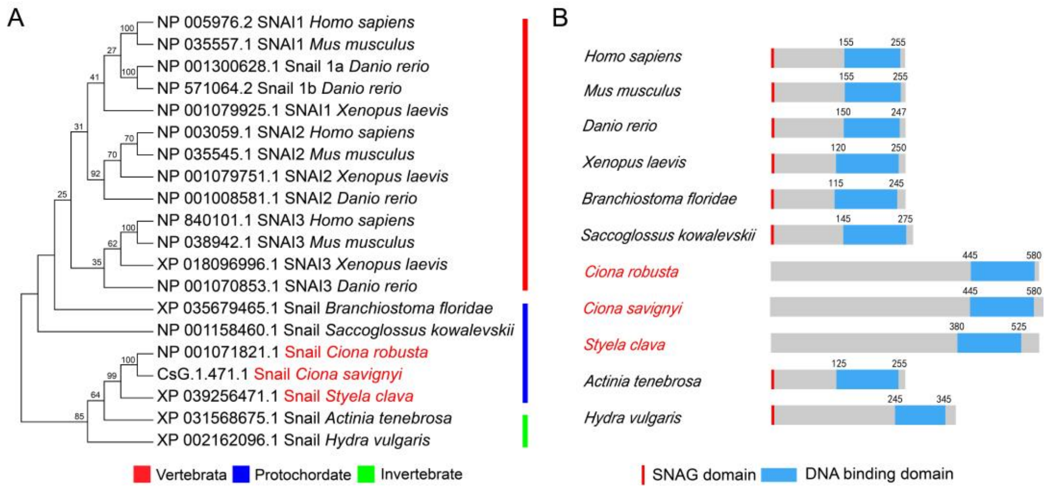

Figure S2. Phylogenetic analysis and structural domain comparison of *Ci-Snail*. (**A**) Phylogenetic analysis by maximum likelihood, bootstraps are 1000, and the model is JTT + G + I. Different color lines represent different clusters, including vertebrates, protochordates, and invertebrates. Red font represents *Ciona* species. (**B**) Structural diagrams of Snail proteins across species. The blue regions indicate DNA-binding domains and red regions indicate SNAG domains.

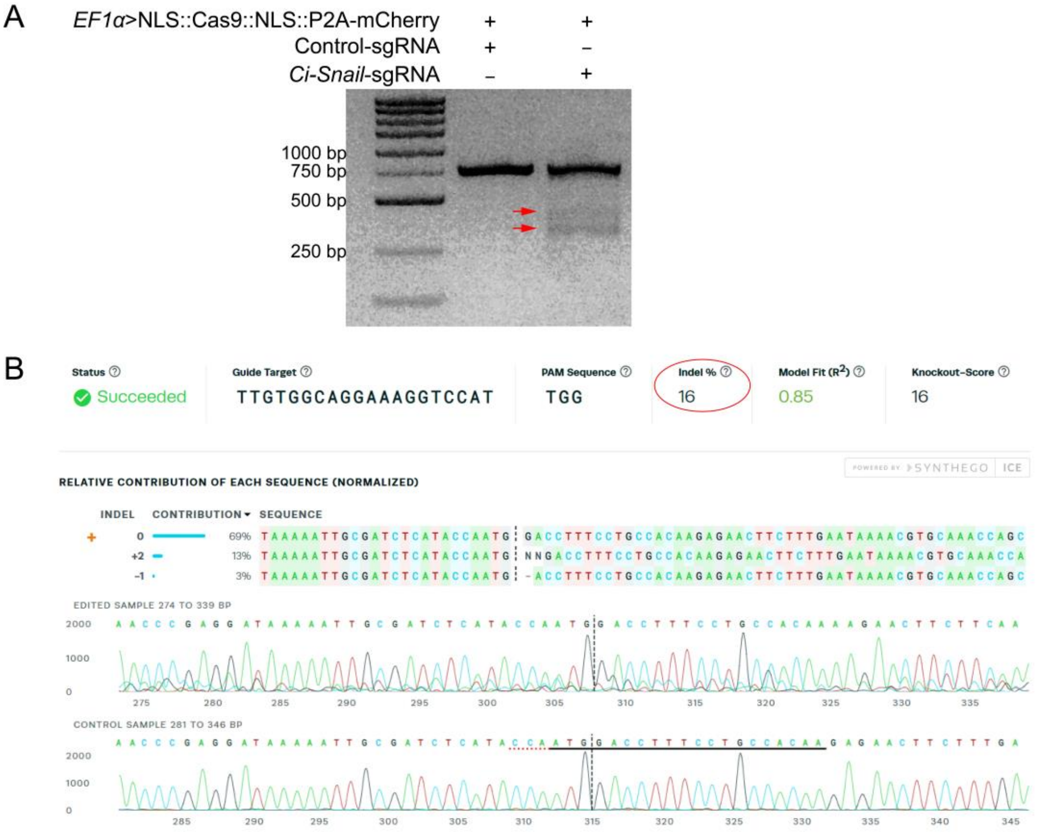

Figure S3. Validation of *Ci-Snail* knockout efficiency. (**A**) T7 Endonuclease assay. The two redundant bands from the *Ci-Snail* KO group were indicated by red arrows. (**B**) Evaluation of the KO efficiency of *Ci-Snail* with Synthego. Red circle highlights the knockout efficiency is 16%, blue line indicates the relative contribution of each sequence, different colored peaks correspond to different colored bases. The vertical black dashed line indicates the actual cleavage site and the horizontal black underlined region indicates the sgRNA sequence. The horizontal red underlined region indicates the PAM site.

Table S1. List of primers.

Primer name	Sequence (5′ - 3′)
KH.L22.27-Promoter-eGFP-F	TCAGATCTCGAGCTCAGCAATGGTACCATAGCAATGCAAC
KH.L22.27-Promoter-eGFP-R	CGCGGTACCGTCGACATTAAATGTTTTGCGTTTATTTGCT
KH.L96.34-Promoter-eGFP-F	TCAGATCTCGAGCTcATATACATTATGTTGTTACATCTAG
KH.L96.34-Promoter-eGFP-R	CGCGGTACCGTCGAcAAGTATTCAACCTTGTACTTTGTAT
KH.L22.27-Promoter-tdTomato-F	TCAGATCTCGAGCTCAGCAATGGTACCATAGCAATGCAAC
KH.L22.27-Promoter-tdTomato-R	CGCGGTACCGTCGACATTAAATGTTTTGCGTTTATTTGCT
KH.L22.27-Brachyury-eGFP-F	GGTACCGCGGGCCCGGGATCCATGACGTCATCAGATAGTAAGTTAG
KH.L22.27-Brachyury-eGFP-R	GGTGGCGACCGGTGCGGATCCCAAAGAAGGTGGCGTAAGCGGCGTA
KH.L22.27-Snail-tdTomato-F	TCGAGCTCAAGCTTCGAATTCATGACCTCCGTCGAGCCCATGCTTT
KH.L22.27-Snail-tdTomato-R	GGTGGCGACCGGTGCGGATCCGGATGCTGTCTTGCGTTGTGCAGTT
KH.L96.34-Brachyury-eGFP-F	GTACCGCGGGCCCGGGATCCATGACGTCATCAGATAGTAA
KH.L96.34-Brachyury-eGFP-R	GTGGCGACCGGTCGGGATCCCAAAGAAGGTGGCGTAAGCG
KH.L22.27-HA-F-1	TCAGATCTCGAGCTCAGCAATGGTACCATAGCAATGCAAC
KH.L22.27-HA-R-1	GGCGTAGTCGGGCACGTCGTAAGGGTAGGTGGCGACCGGTGGATCC
KH.L22.27-HA-F-2	TACCCTTACGACGTGCCCGACTACGCCAGCGGCCGCGACTCTAGA
KH.L22.27-HA-R-2	TATGGTACCATTGCTGAGCTCGAGATCTGAGTCCGGTAGC
KH.L22.27-Snail-HA-F	GGTACCGCGGGCCCGGGATCCATGACCTCCGTCGAGCCCATGCTTT
KH.L22.27-Snail-HA-R	GGTGGCGACCGGTGCGGATCCGGATGCTGTCTTGCGTTGTGCAGTT
U6 forward-sgRNA-F	AGATTGGCGGGTGTATTAAACCAC
Reverse-sgRNA-R	AAACGGATTTCCTTACGCGAAATACG
Ci-Snail-sgRNA-F	AGATGTTGTGGCAGGAAAGGTCCAT
Ci-Snail-sgRNA-R	AAACATGGACCTTTCCTGCCACAAC
Ci-Snail-sgRNA-test-F	TACAGACACAGTGATGAATACAAAACTGATACCACCTCAT
Ci-Snail-sgRNA-test-R	TAGTTGAGCGAATTAGTAGTTGGTACTGACGAACATGACG
Ci-Snail-qPCR-F	CAGAACCGATTGATGACTA
Ci-Snail-qPCR-R	TTGAAGTTATTGGAGTGGAA
Brachyury-qPCR-F	CAGATAGTAAGTTAGCAGGTA
Brachyury-qPCR-R	AATACATCGCAGTTGGAT
Internal reference-U6-qPCR-F	TGTACTTGCTTCGGCAGTACATA
Internal reference-U6-qPCR-R	AGGAACGCTTCACGATTTTG
Snail binding site-F	AACCTTATCTGGTGTTACG
Snail binding site-R	GTTACTTTCCCATTGTCTTG

Table S2. List of Snail family members across species.

Species	Gene	ID	Amino acid length (aa)
Homo sapiens	SNAI1	NP_005976.2	264
	SNAI2	NP_003059.1	268
	SNAI3	NP_840101.1	292
Mus musculus	SNAI1	NP_035557.1	264
	SNAI2	NP_035545.1	269
	SNAI3	NP_038942.1	287
Danio rerio	SNAI1a	NP_001300628.1	260
	SNAI1b	NP_571064.2	256
	SNAI2	NP_001008581.1	257
	SNAI3	NP_001070853.1	283
Xenopus laevis	SNAI1	NP_001079925.1	259
	SNAI2	NP_001079751.1	266
	SNAI3	XP_018096996.1	281
Branchiostoma floridae	Snail	XP_035679465.1	253
Saccoglossus kowalevskii	Snail	NP_001158460.1	287
Ciona Robusta	Snail	NP_001071821.1	585
Ciona savignyi	Snail	CsG.1.471.1	597
Styela clava	Snail	XP_039256471.1	581
Actinia tenebrosa	Snail	XP_031568675.1	265
Hydra vulgaris	Snail	XP_002162096.1	350
